# Impact of the COVID-19 pandemic and the dynamic COVID-zero strategy on HIV incidence and mortality in China

**DOI:** 10.1186/s12889-023-15268-9

**Published:** 2023-02-18

**Authors:** Lan Wang, Na Zhao, Yuliang Wang, Kaili Sun, Yike Wang, Shufang Huang, Feng Yao, Xiangyu Guo, Yunmei Yang, Chenjin Ma, Shelan Liu

**Affiliations:** 1 Department of Geriatrics, the First Affiliated Hospital, Zhejiang University School of Medicine, Hangzhou, 310003 China; 2 Key Laboratory of Diagnosis and Treatment of Aging and Physic-chemical Injury Diseases of Zhejiang Province, Hangzhou, 310003 China; 3 School of Ecology and Environment, Anhui Normal University, Wuhu, 241002 Anhui Province China; 4 Department of Immunology, Basic Medical School, Nanjing Medical University, Nanjing, 211166 China; 5 College of Statistics and Data Science, Faculty of Science, Beijing University of Technology, Beijing, 100124 China; 6 School of Public Health, Guangdong Pharmaceutical University, Guangzhou, 510315 Guangdong Province China; 7 Peking University Health Science Center, Beijing, 100191 China; 8 Department of Neuro-Oncology, Cancer Center, Beijing Tiantan Hospital, Capital Medical University, Beijing, 100050 China; 9 Department of Infectious Diseases, Zhejiang Provincial Center for Disease Control and Prevention, Hangzhou, 310051 Zhejiang Province China

**Keywords:** COVID-19, HIV, Incidence, Mortality, Non-pharmaceutical interventions, China

## Abstract

**Background:**

In response to the coronavirus disease 2019 (COVID-19) pandemic, the Chinese government implemented the *dynamic COVID-zero strategy*. We hypothesized that pandemic mitigation measures might have reduced the incidence, mortality rates, and case fatality ratios (CFRs) of the human immunodeficiency virus (HIV) in 2020–2022.

**Method:**

We collected HIV incidence and mortality data from the website of the National Health Commission of the People’s Republic of China from January 2015 to December 2022. We compared the observed and predicted HIV values in 2020–2022 with those in 2015–2019 using a two-ratio Z-test.

**Results:**

From January 1, 2015, to December 31, 2022, a total of 480,747 HIV incident cases were reported in mainland China, of which 60,906 (per year) and 58,739 (per year) were reported in 2015–2019 (pre-COVID-19 stage) and 2020–2022 (post-COVID-19 stage), respectively. The average yearly HIV incidence decreased by 5.2450% (from 4.4143 to 4.1827 per 100,000 people, *p* <  0.001) in 2020–2022 compared with that in 2015–2019. However, the average yearly HIV mortality rates and CFRs increased by 14.1076 and 20.4238%, respectively (all *p* <  0.001), in 2020–2022 compared with those in 2015–2019. During the emergency phase in January 2020 to April 2020, the monthly incidence was significantly lower (23.7158%) than that during the corresponding period in 2015–2019, while the incidence during the routine stage in May 2020–December 2022 increased by 27.4334%, (all *p* <  0.001). The observed incidence and mortality rates for HIV decreased by 16.55 and 18.1052% in 2020, by 25.1274 and 20.2136% in 2021, and by 39.7921 and 31.7535% in 2022, respectively, compared with the predicted values, (all *p* <  0.001).

**Conclusions:**

The findings suggest that China’s dynamic COVID-zero strategy may have partly disrupted HIV transmission and further slowed down its growth. Without China’s dynamic COVID-zero strategy, HIV incidence and deaths in the country would have likely remained high in 2020–2022. There is an urgent need to expand and improve HIV prevention, care, and treatment, as well as surveillance in the future.

**Supplementary Information:**

The online version contains supplementary material available at 10.1186/s12889-023-15268-9.

## Background

The first case of coronavirus disease 2019 (COVID-19) was identified in the city of Wuhan in Hubei Province, China, in December 2019 [1–4]. On March 11, 2020, the World Health Organization declared the novel coronavirus outbreak a global pandemic [5, 6]. The number of confirmed global COVID-19 cases at the time of writing on January 20, 2023, had exceeded 663 million, and 6.71 million deaths had been reported in more than 220 countries, areas, and territories; of this total, 11,001,439 confirmed cases and 34,375 deaths were reported in China [7]. The COVID-19 outbreak has adversely affected national economies and the health and lifestyles of people throughout the world. Without a doubt, the COVID-19 pandemic is the worst public threat that humans have encountered in the past 100 years [8]. In response to it, most countries have implemented a range of social and physical distancing measures and have administered severe acute respiratory syndrome coronavirus-2 vaccines to reduce disease transmission [9]. These measures and the redeployment of health staff as part of the pandemic response have been gradually applied, reducing or reconfiguring the clinical services providing care for other infectious diseases, including sexually transmitted infections [10].

Previous studies have shown that the COVID-19 pandemic and the responses to this threat have resulted in a substantial decline in the number of human immunodeficiency virus (HIV)-infected individuals in many countries, with significant undertesting, underreporting, and underservice [10]. A survey conducted in 19 countries in Central and Eastern Europe reported that HIV clinics normally operated in only six of these countries (∼32%) during the pandemic [11–13]. A study reported the reduced initiation of HIV treatment in South Africa during the pandemic [14]. Another study from Italy, Spain, and Taiwan suggested that decreases in HIV cases were also observed [15]. However, no study of HIV epidemiology during the COVID-19 pandemic has been reported in China, where HIV is one of the most severe disease burdens [16].

COVID-19 hit China in early December 2020. The entire country experienced the large-scale epidemic in the first season of 2020, which was named the *emergency response stage*. Since May 2020, China has entered the *normalization stage* of prevention and control [17]. In response to the spread of the highly transmissible Delta variant, China adopted a new strategy called *dynamic zero-COVID* in August 2021, which successfully controlled the spread of the disease at a lower cost and in a shorter time [18]. This measure differed considerably from the suppression and mitigation strategies applied in other countries. In response to the pandemic, most hospitals in China diverted their healthcare resources to the outbreak, limiting services for other medical conditions, including HIV/acquired immunodeficiency syndrome (AIDS). Although a few articles have discussed the potential impact of COVID-19 restrictions on HIV in Guangxi Province, we are not aware of any empirical study that has estimated the actual changes in the number of deaths and cases among a large sample at the national level [13]. Therefore, the current report presents analyses of national surveillance data to assess the impact of the COVID-19 pandemic and the dynamic COVID-zero strategy on HIV incidence, mortality rates, and case fatality ratios (CFRs) in China. This research will help healthcare providers navigate shifting tasks and reset priorities effectively while developing clear guidelines to best serve HIV patients after the pandemic.

## Methods

### Data sources

Since 2004, China has used the direct network reporting system called the China Infectious Disease Reporting Information System [19]. Diseases are divided into three categories—classes A (two diseases), B (27 diseases), and C (11 diseases)—all of which must be reported within specified timeframes of 2 hours, 24 hours, and 24 hours, respectively [19]. HIV cases must be identified as Class B, a notifiable infectious disease.

In this study, data on monthly HIV cases and deaths, including confirmed cases that occurred from January 2015 to December 2022, were obtained from the National Health Commission of the People’s Republic of China, which releases a monthly report of notifiable infectious diseases online. Chinese population data from 2015 to 2022 were taken from the National Bureau of Statistics. Monthly and annual incidences or mortality rates for HIV (per 100,000 people) were defined as the number of monthly and annual cases or deaths divided by the population size. The CFR is calculated by dividing the number of deaths from HIV during a defined period of time by the number of individuals diagnosed with HIV during that time; the resulting ratio is then multiplied by 100 to yield a percentage.

### Case definition

The criteria for confirmed HIV cases were defined according to *the Chinese Guidelines for the Diagnosis and Treatment of HIV/AIDS* (2021 Edition), which was published in December 2021 [20]. All reported cases were reviewed by epidemiologists from local centers for disease control to ensure the data’s completeness and validity.

### Interrupted time series

China adopted its *zero-infections policy* to eliminate COVID-19 in two stages: January 2020–April 2020, which was identified as the emergency stage of COVID-19, and May 2020 to date, which was identified as the routine stage, of which August 2021–December 2022 was identified as the dynamic COVID-zero stage [17, 18]. The current study compares the monthly incidence, mortality rates, and CFRs of HIV diseases during the pre-emergency stage, emergency stage, and post-emergency stage (routine stage).

### Statistical analysis

To visually demonstrate the effects of COVID-19 on HIV incidence, mortality rates, and CFRs, we categorized the data according to time of occurrence: 2015–2019 (pre-COVID-19) and 2020–2022 (post-COVID).

During the data analysis, summary statistics were applied for HIV. Specifically, the following averages were computed: annual number of cases, annual incidence, monthly incidence, annual number of deaths, annual mortality, and monthly mortality.

We defined yearly/monthly incidence (per 100,000) as the number of yearly/monthly incident cases divided by the total population size, overall, yearly/monthly mortality (per 100,000) as the number of yearly/monthly deaths divided by the total population size, and case fatality ratio (per 100) as the number of yearly/monthly deaths divided by the number of yearly/monthly incident cases. More details are presented in the following:

#### Pre-COVID-19 stage

1. Average yearly incidence = (total number of new cases from 2015 to 2019 / 5) / (sum of the population from 2015 to 2019 / 5) * 100,000
2. Average monthly incidence = (total number of new cases from 2015 to 2019 / (5 * 12)) / (sum of the population from 2015 to 2019 / 5) * 100,000
3. Average yearly mortality = (total number of new deaths from 2015 to 2019 / 5) / (sum of the population from 2015 to 2019 / 5) * 100,000
4. Average monthly mortality = (total number of new deaths from 2015 to 2019 / (5 * 12)) / (sum of the population from 2015 to 2019 / 5) * 100,000
5. Average yearly CFR = (total number of deaths from 2015 to 2019 / 5) / (total number of cases from 2015 to 2019 / 5) * 100
6. Average monthly CFR = (total number of deaths from 2015 to 2019 / (5 * 12)) / (total number of cases from 2015 to 2019 / (5 * 12)) * 100

#### Post-COVID-19 stage

1. Average yearly incidence = (total number of new cases from 2020 to 2022 / 3) / (sum of the population from 2020 to 2022 / 3) * 100,000
2. Average monthly incidence = (total number of new cases from 2020 to 2022 / (3 * 12)) / (sum of the population from 2020 to 2022 / 3) * 100,000
3. Average yearly mortality = (total number of new deaths from 2020 to 2022 / 3) / (sum of the population from 2020 to 2022 / 3) * 100,000
4. Average monthly mortality = (total number of new deaths from 2020 to 2022 / (3 * 12)) / (sum of the population from 2020 to 2022 / 3) * 100,000
5. Average yearly CFR = (total number of deaths from 2020 to 2022 / 3) / (total number of cases from 2020 to 2022 / 3) * 100
6. Average monthly CFR = (total number of deaths from 2020 to 2022 / (3 * 12)) / (total number of cases from 2020 to 2022 / (3 * 12)) * 100

We applied two proportional tests to analyze changes in the average yearly incidence, yearly mortality rates, and CFRs for 2015–2022. Changes were computed to identify the monthly incidence, monthly mortality rates, and CFR growth rates. A two-ratio Z-test was used to examine the differences in monthly incidence, mortality rates, and CFRs in the emergency phase and in the routine phase between 2015 and 2019 and 2020–2022. We used the autoregressive integrated moving average (ARIMA) model to predict the incidence, mortality rates, and CFRs from 2020 to 2022 on the basis of 2015–2019 HIV data. The ARIMA model has shown good predictive properties in many statistical and medical studies. A two-ratio Z-test was also used to study the differences between the observed and predicted values. The Wilcoxon rank test was applied to examine the correlation between the number of HIV cases monthly and the number of COVID-19 cases monthly. A regression line was also fitted between the number of HIV cases monthly and the number of COVID-19 cases monthly. We computed the lower and upper 95% confidence intervals. In all tests, a *p*-value cut-off of 0.05 was used.

### Patient and public involvement

Involving patients or the public in the design, conduct, reporting, or dissemination plans of our research was not appropriate or possible.

## Results

### Overall yearly incidence trend

From January 1, 2015, to December 31, 2022, a total of 480,747 HIV cases were reported in mainland China, of which 60,906 (per year) and 58,739 (per year) were reported in 2015–2019 (pre-COVID-19 stage) and 2020–2022 (post-COVID-19 stage), respectively.

During the post-COVID-19 stage, a total of 63,154, 61,032, and 52,032 new cases were reported in 2020, 2021, and 2022, respectively. The average yearly HIV incidence decreased by 5.2450% (from 4.4143 to 4.1827 per 100,000 people, *p* <  0.001) in 2020–2022 compared with that in 2015–2019 (Table 1).

**Table 1:** Comparisons of the average yearly HIV incidence, mortality rates, and case fatality rates between 2020 and 2022 and 2015–2019

Variable	Post-COVID-19 Stage (2020–2022)	Pre-COVID-19 Stage (2015–2019)	Changes (%)	95% CI	***p-***value
Average Yearly Incidence Rate (/100,000)	4.1827	4.4143	−5.2450	−5.2766, − 5.2154	< 0.001
Average Yearly Mortality Rate (/100,000)	1.3773	1.2070	14.1076	14.0430, 14.1877	< 0.001
Average Yearly Case Fatality Rate (/100)	32.9285	27.3439	20.4238	20.3842, 20.4629	< 0.001

Changes = (x_2_ – x_1_) / x_1_ × 100, x1: average yearly incidence/mortality/CFR in 2015–2019; x2: average yearly incidence/mortality/CFR in 2020–2022. *p*-values were computed using two proportional tests

Pre-COVID-19 Stage:

(1) Average yearly incidence = (total number of new cases from 2015 to 2019 / 5) / (sum of the population from 2015 to 2019 / 5) * 100,000

(2) Average yearly mortality = (total number of new deaths from 2015 to 2019 / 5) / (sum of the population from 2015 to 2019 / 5) * 100,000

(3) Average yearly CFR = (total number of deaths from 2015 to 2019 / 5) / (total number of cases from 2015 to 2019 / 5) * 100

Post-COVID-19 Stage:

(1) Average yearly incidence = (total number of new cases from 2020 to 2022 / 3) / (sum of the population from 2020 to 2022 / 3) * 100,000

(2) Average yearly mortality = (total number of new deaths from 2020 to 2022 / 3) / (sum of the population from 2020 to 2022 / 3) * 100,000

(3) Average yearly CFR = (total number of deaths from 2020 to 2022 / 3) / (total number of cases from 2020 to 2022 / 3) * 100

### Overall yearly mortality rate and CFR trends

From January 1, 2015, to December 31, 2022, a total of 141,296 HIV deaths were reported in China, of which 16,654 (per year) and 19,342 (per year) were reported in 2015–2019 and 2020–2022, respectively. In the post-COVID-19 stage, a total of 17,056, 20,259, and 18,788 newly reported deaths were collected in 2020, 2021, and 2022, respectively.

The average yearly HIV mortality rates increased by 14.1076% (from 1.2070 to 1.3773 per 100,000 people, *p* <  0.001) in 2020–2022 compared with those in 2015–2019 (Table 1). Between 2020 and 2022 and 2015–2019, the average yearly HIV CFRs increased by 20.4238% (from 27.3439 to 32.9285 per 100 people, *p* <  0.001; Table 1).

### Monthly HIV incidence in the pre-emergency, emergency, and post-emergency periods (routine phase)

**Fig. 1:**
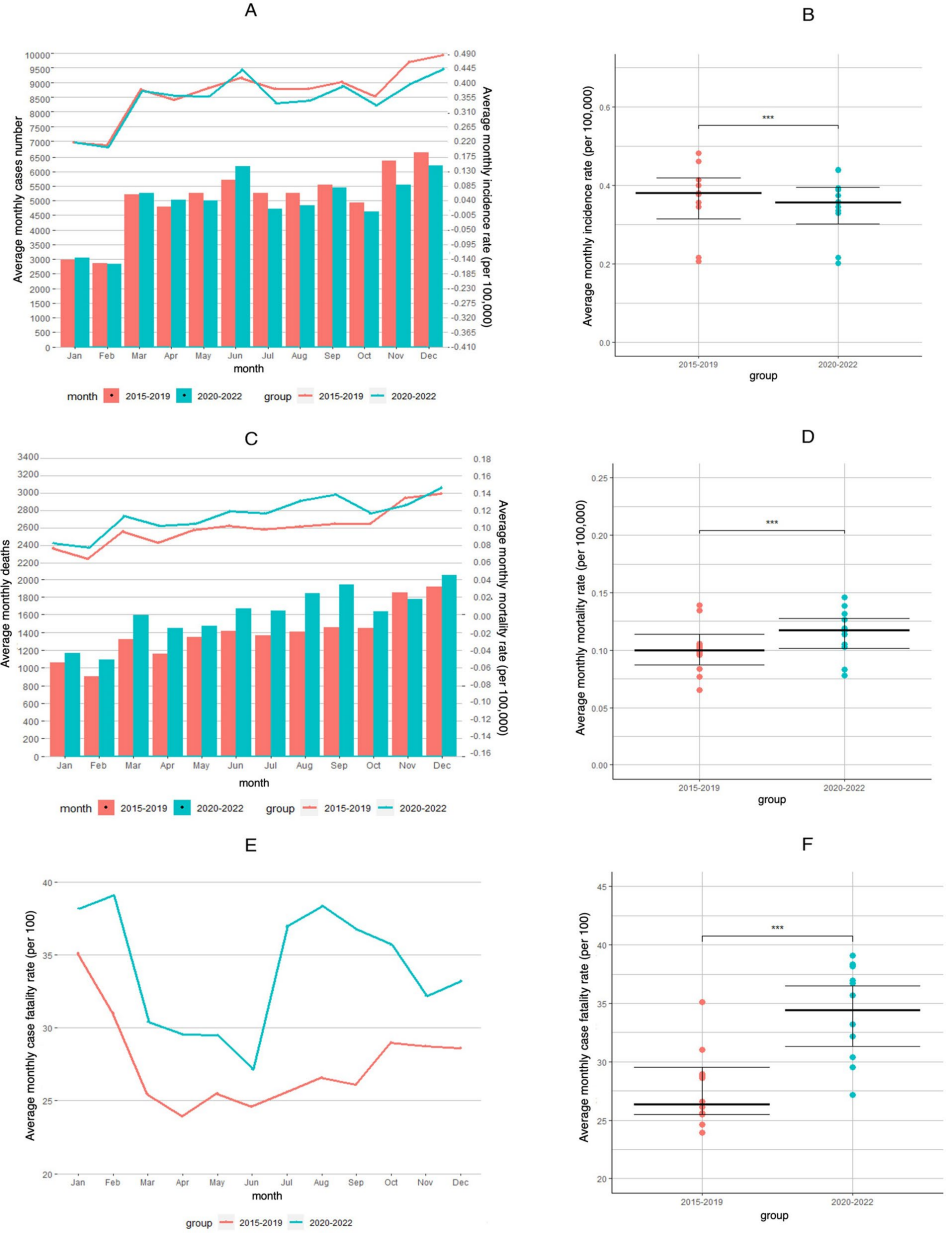
Trends for the monthly incidence, mortality rates, and CFRs for HIV in China from 2015 to 2022. Notes: **A** Total number of HIV cases reported monthly and the monthly incidence of HIV; **C** Total number of HIV deaths reported monthly and the monthly mortality rates of HIV; **E** Monthly CFRs; 2015–2019 (red bar and red line) and 2020–2022 (blue bar and blue line). **B**, **D**, and **F** Comparisons of the yearly incidence, mortality rates, and CFRs for HIV between 2015 and 2019 and 2020–2022 (***, *p* <  0.001; **, 0.001 < *p* <  0.01; *, 0.01 < *p* <  0.05). Black lines indicate P25, P50, and P75 for the upper and lower quartiles. Dots indicate the monthly incidence, mortality rates, and case fatality rates from January to December in different years

The studied periods were further stratified by three phases: pre-emergency (2015–2019), emergency (January 2020–April 2020), and post-emergency (routine phase, from May 2020 to December 2022). The average monthly HIV incidence during the emergency phase was lower than that during the pre-emergency period (0.2803 vs. 0.3675 per 100,000 people, a 23.7158% decrease, *p* <  0.001); Fig. 1A and B; Table 2). By contrast, the average monthly incidence during the routine phase was considerably higher than that during the emergency period (0.3572 vs. 0.2803 per 100,000 people, a 27.4334% increase, *p* <  0.001; Fig. 1A and B, Table 2).

**Table 2:** Changes in the average monthly HIV incidence, mortality rates, and case fatality rates in the pre-emergency, emergency, and post-emergency periods (routine phases) in China

Variable	Pre-Emergency Stage (January 2015–December 2019)	Emergency Stage (January 2020–April 2020)	Post-Emergency Stage (Routine Stage) (May 2020–December 2022)	Changes 1 (%)	P1-value	Changes 2 (%)	P2-value
	X1	X2	X3	X2 vs. X1		X3 vs. X2	
Monthly Incidence Rate (/100,000)	0.3675	0.2803	0.3572	−23.7158	< 0.001	27.4334	< 0.001
Monthly Mortality Rate (/100,000)	0.1004	0.0882	0.1181	−12.1606	< 0.001	33.8750	< 0.001
Monthly Case Fatality Rate (/100)	27.3290	31.4687	33.0594	15.1475	< 0.001	5.0549	< 0.001

(1) X1 = monthly incidence/mortality/CFR in the pre-emergency stage (January 2015–December 2019)

(2) X2 = monthly incidence/mortality/CFR in the emergency stage (January 2020–April 2020)

(3) X3 = monthly incidence/mortality/CFR in the post-emergency stage (May 2020–December 2022)

(4) Changes 1 = (X2 − X1) / X1 × 100

(5) Changes 2 = (X3 – X2) / X2 × 100

(6) The *p*-values for X2 vs. X1 and X3 vs. X2 were computed using two-ratio Z-tests

(7) The calculated formula:

Pre-emergency stage (January 2015–December 2019)

Average monthly incidence = (total number of new cases from 2015 to 2019 / (5 * 12)) / (sum of the population from 2015 to 2019 / 5) * 100,000

Average monthly mortality = (total number of new deaths from 2015 to 2019 / (5 * 12)) / (sum of the population from 2015 to 2019 / 5) * 100,000

Average monthly CFR = (total number of deaths from 2015 to 2019 / (5 * 12)) / (total number of cases from 2015 to 2019 / (5 * 12)) * 100

Emergency stage (January 2020–April 2020)

Average monthly incidence = (total number of new cases from January 2020 to April 2020 / 4 / (sum of the population from January 2020 to April 2020/4) * 100,000

Average monthly mortality = (total number of new deaths from January 2020 to April 2020 / 4 / sum of the population from January 2020 to April 2020/4) * 100,000

Average monthly CFR = (total number of deaths from January 2020 to April 2020 /4 / (total number of cases from January 2020 to April 2020/4) * 100

Post-emergency period (routine phases)

Average monthly incidence = (total number of new cases from May 2020 to December 2022 / 32 / (sum of the population from May 2020 to December December 2022 / 32) * 100,000

Average monthly mortality = (total number of new deaths from May 2020 to December 2022 / 32 / (sum of the population from 2020 to December 2022 / 32) * 100,000

Average monthly CFR = (total number of deaths from May 2020 to December 2022/ 32 / (total number of cases from May 2020 to December 2022 / 32) * 100

The monthly HIV incidence for 2015–2022 is presented in Supplementary Fig. 1. The scatter diagram of the number of reported HIV cases monthly and the number of reported COVID-19 cases monthly from 2020 to 2022 is presented in Supplementary Fig. 2. We can see from the fitting line that the number of HIV cases and the number of COVID-19 cases have a negative correlation (*p* <  0.001).

### Monthly HIV mortality rates in the pre-emergency, emergency, and post-emergency periods (routine phase)

In 2020, the average monthly HIV mortality rates during the emergency phase (January–April) were lowerer than that in 2015–2019 (0.0882 vs. 0.1004 per 100,000 people, a 12.1606% decrease, *p* <  0.001; Fig. 1C and D; Table 2). Moreover, the average monthly mortality rates during the routine phase increased by 33.8750% (from 0.0882 to 0.1181 per 100,000 people, *p* <  0.001) compared with those during the emergency period (Fig. 1C and D; Table 2).

### Monthly HIV CFRs in the pre-emergency, emergency, and post-emergency periods (routine phase)

In 2020, the average monthly HIV CFRs increased by 15.1475% (from 27.3290 to 31.4687 per 100 people, *p* <  0.001) during the emergency phase (January–April) compared with those during the corresponding period in 2015–2019. Meanwhile, the average monthly CFRs during the routine phase in May 2020–December 2022 further increased by 5.0549% (from 31.4687 to 33.0594 per 100, *p* <  0.001) compared with those during the emergency period in 2020 (Fig. 1E and F; Table 2).

### Observed and predicted monthly incidence, mortality rates, and CFRs

**Fig. 2:**
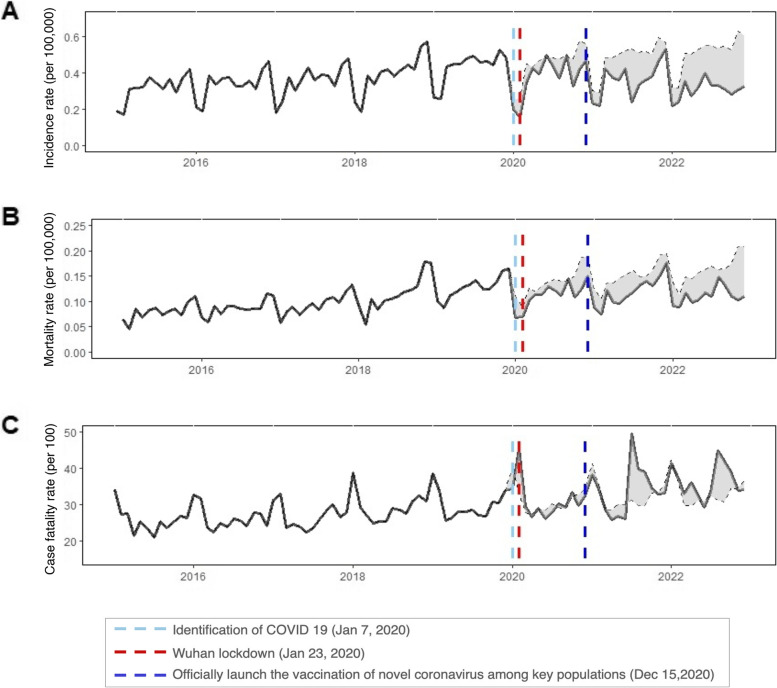
Observed (black lines) and predicted (black dashed lines) HIV values from 2015 to 2022 in China. Notes: **A** HIV incidence. **B** HIV mortality rates. **C** CFRs for HIV

The observed yearly HIV incidence was significantly lower than the predicted values in 2020 (16.55% lower), 2021 (25.1274% lower), and 2022 (39.7921% lower), all *p* <  0.001 (Fig. 2; Table 3).

**Table 3:** Observed and predicted HIV incidence, mortality rates, and case fatality rates in China in 2020, 2021, and 2022

**Variables**	**2020**				
	**Observed**	**Predicted**	**Changes 1 (%)**	**95% CI**	***p***-**value**
Incidence Rate (/100,000)	0.3768	0.4516	−16.5500	−16.5888, − 16.5378	< 0.001
Mortality Rate (/100,000)	0.1133	0.1383	−18.1052	−18.1329, − 18.0204	< 0.001
Case Fatality Rate (/100)	30.0519	31.1409	−3.4968	−3.5316, 3.4624	< 0.001
	**2021**				
	**Observed**	**Predicted**	**Changes 2 (%)**	**95% CI**	***p*-value**
Incidence Rate (/100,000)	0.3623	0.4839	−25.1274	−25.1529, − 25.1055	< 0.001
Mortality Rate (/100,000)	0.1203	0.1507	−20.2136	−20.2256, − 20.1195	< 0.001
Case Fatality Rate (/100)	33.1941	32.1872	3.1280	3.0942, 3.1624	< 0.001
	**2022**				
	**Observed**	**Predicted**	**Changes 3 (%)**	**95% CI**	***p*-value**
Incidence Rate (/100,000)	0.3070	0.5098	−39.7921	−39.8023, − 39.7583	< 0.001
Mortality Rate (/100,000)	0.1108	0.1624	−31.7535	− 31.8226, − 31.7242	< 0.001
Case Fatality Rate (/100)	36.1085	33.3384	8.3091	8.2758, 8.3424	< 0.001

Changes 1 = (Observed − Predicted) / Predicted × 100 in 2020; Changes 2 = (Observed − Predicted) / Predicted × 100 in 2021; Changes 3 = (Observed − Predicted) / Predicted × 100 in 2022; *p-*values were computed using two proportional tests. The *p-*values for the emergency phase versus the routine response phase were computed using two-ratio Z-tests

The observed mortality rates were much lower than the predicted values in 2020 (an 18.1052% reduction), 2021 (a 20.2136% reduction), and 2022 (a 31.7535% reduction), all *p* <  0.001 (see Fig. 2; Table 3).

The observed CFR was slightly lower than the predicted value in 2020 (a 3.4968% reduction), but it rose by 3.1280% in 2021 and further increased by 8.3091% in 2022, all *p* <  0.001 (see Fig. 2; Table 3).

## Discussion

Before the COVID-19 pandemic, the HIV situation was a major public health concern in China. In this study, we hypothesized that pandemic mitigation measures might have influenced HIV incidence, mortality rates and CFR. National surveillance data from 31 provinces showed a slight decrease in the overall incidence and number of HIV cases during the COVID-19 pandemic in 2020–2022 compared to the pre-COVID-19 period (2015–2019). Gradual increases in HIV mortality rates and CFRs were observed in 2020–2022 compared to the five previous years. By contrast, the observed incidence and mortality rates in 2020–2022 were much lower than predicted. These findings suggest that China’s dynamic COVID-zero strategy may have helped slow down HIV transmission. The strength of this study is its largest sample size for 2015–2022, which cover all 31 provinces and have been analyzed at the national level.

The first HIV infection was identified in China 35 years ago [21, 22]. Newly reported HIV/AIDS cases in China increased annually by 15 times, that is, from 2705 cases in 2005 to 42,406 cases in 2019 [16]. Meanwhile, the number of HIV/AIDS-related deaths only increased by 25%, that is, from 40,711 in 2005 to 51,250 in 2019, despite China’s substantial progress in HIV screening and therapy [23]. HIV transmission in China has evolved from substance abuse via injections to outbreaks as a result of plasma collection contamination in the mid-1990s and to the almost exclusive transmission via sexual contact at present [24]. Declines in HIV cases and diagnoses after the implementation of COVID-19 restrictions have been reported across Europe and elsewhere [10, 25, 26], ,but HIV-specific data on this phenomenon are lacking in China. Our results show that the overall yearly HIV incidence decreased by 5.2450% in 2020–2022 compared with that in 2015–2019. Specifically, the monthly incidence during the emergency phase of COVID-19 in 2020 was significantly lower than that during the corresponding period in the five previous years. This decline was due to several reasons [27]. First, strict quarantine measures, the prohibition of public gatherings, and the closure of public cultural and entertainment venues may have drastically reduced opportunities to travel and meet sexual partners, thus reducing the risk of exposure during this period [28]. Second, the response to the COVID-19 pandemic in 2020–2022 likely led to underdiagnosis of HIV in China, including the disruptions in clinical care services, shortages in HIV testing reagents and materials, shifting of partner services staff to COVID-19 activities [10]. Third, HIV consultations and diagnoses likely decreased; there were delays in and/or avoidance of urgent or emergency and routine medical care because of COVID-19 concerns [29, 30]. By contrast, the partial increase since the summer of 2020 may be explained by the following three causes. First, sexual behavior rebounded while service interruption persisted, and cases will increase by the hundreds for HIV and by the thousands for COVID-19, which aligns with a report from New York [10]. Second, undetected HIV transmission during the emergency stage will only lead to further increases in transmission [31]. Third, 40–90% of people have flu-like symptoms within two to 4 weeks after HIV infection, which are somewhat similar to the early symptoms of COVID-19 infection [32]. Most HIV patients have had more cause to seek medical care out of fear of COVID-19 during the routine stage [33].

Our study showed that HIV-related deaths in China in 2020 and 2022 continuously increased compared to those 2015–2019. In the country, many of the primary healthcare activities related to the long-term management of HIV are undertaken mainly at hospitals [34]. During the COVID-19 outbreak, access to essential, life-saving HIV drugs through hospital visits was severely compromised, exacerbating established HIV cases and increasing the risk of death. Most importantly, HIV carriers were not diagnosed early because they were worried about contracting COVID-19 at the hospital. Second, people living with HIV (PLWH) cannot refill their medication because of a shortage in antiretroviral therapy (ART) drugs and strict quarantine measures and transportation lockdowns. Disruptions in HIV treatment provisions are concerning because direct-acting antivirals clear the virus, so their inaccessibility increases the potential for transmission and death. The Joint United Nations Programme on HIV/AIDS and the BaiHuaL, in alliance with PLWH and with the support of the Chinese National Center for AIDS/STD Control and Prevention, surveyed PLWH in China in February 2020. Of this population, 32.6% were found to be at risk of ART discontinuation, and about 48.6% did not know where to get antiviral drugs in the near future [35, 36]. Third, during the COVID-19 epidemic, many healthcare facilities were diverted into caring for patients with COVID-19. PLWH who should have started with ART might have been deterred or delayed. Fourth, PLWH not only experience a deterioration of their physical health but also suffer great psychological and economic pressure [37]. They face a higher risk of COVID-19 deaths than people without HIV after adjusting for age and sex (hazard ratio: 2.90) [38, 39].

Consistent with previous reports in China and elsewhere, the observed total HIV incidence, mortality rates were significantly lower than the predicted rates in 2020–2022. This finding suggests that China’s the dynamic COVID-zero strategy in 2020–2022 significantly decreased the overall incidence and mortality rates of HIV in China [34]. Strict containment and suppression strategies changed people’s behaviors and sexual relationships. Meanwhile, these nonpharmaceutical interventions (NPIs) also resulted in difficulties in accessing hospital services and reluctance to seek hospital care during the outbreak, hence the fewer HIV diagnoses and patient notifications made [10]. During China’s expansion phase (1995 to the present), HIV has been characterized by an increasing prevalence and expanded geographic reach. Since then, the number of HIV/AIDS cases has grown rapidly [16, 40]. The expected incidence and mortality in 2020 and 2022 increased, which might have partially resulted from the long-term trend of HIV diseases in China [41].

### Limitations

This study has the following limitations. First, the COVID-19 pandemic in China led to disruptions in HIV testing services and access to clinical services in 2020–2022. There was a steep decrease in HIV diagnoses, mostly attributed to declines in testing caused by less frequent visits to health centers, reduced outreach services, and the shifting of public health staff to COVID-19 response efforts. This likely influenced the underreporting of the yearly incidence and mortality rates of COVID-19. Second, a potential data bias in incidence and mortality rates was reported in different provinces because of the intensity of the epidemic, its scope, and the measures taken, which varied from province to province. Third, this study did not compare referrals in 2020–2022 to previous data on age- and gender-specific populations, resulting in the failure to accurately predict the effects on high-risk populations under the dynamic COVID-zero strategy. Fourth, the changes in the past 8 years can be attributed to economic development and advances in medical health technology. In 2003, China rolled out its key policy of Four Frees, One Care in HIV/AIDS prevention and control and strengthened almost two decades of well-funded and comprehensive response efforts in HIV prevention and control. The diagnosis technology for HIV diseases has also been improved in recent decades, particularly laboratory detection and case identification at all provincial levels. Likewise, pre- or post-exposure prophylaxis has been widely applied in recent years after a single high-risk event to stop HIV seroconversion.

## Conclusion

The nationwide surveillance of HIV cases in China showed that the HIV cases and incidence decreased slightly in 2020–2022 compared with that in 2015–2019, while the mortality rates and CFRs during the two periods increased. Further stratified by phases, the HIV incidence in the emergency stage of 2020 greatly decreased compared with that in the corresponding period of the five previous years, while it has obviously rebounded in post-emergency period. All observed incidence and mortality rates in 2020–2022 remained below the predicted levels. The findings of this study suggest that China’s zero-COVID strategy may have partly disrupted HIV transmission and further slowed down its growth. As the COVID-19 pandemic is still ongoing, more time and data are needed to assess accurately the effects of China’s zero-COVID strategy on HIV infection transmission and long-term outcomes in the country.

## Supplementary Information

**Additional file 1: Supplementary Figure 1.** Trends in the monthly incidence, mortality rates, and CFRs for HIV in China in 2015–2019, 2020, 2021, and 2022. **Supplementary Figure 2. **Scatter plot of the number of monthly reported HIV cases and the number of monthly reported COVID-19 cases in 2020–2022, China.

## Data Availability

The data used in this article can be made available by the corresponding author upon request.

## Abbreviations

ART: Antiretroviral therapy
CFR: Case fatality ratio
COVID-19: Coronavirus disease 2019
HIV: Human immunodeficiency virus
PLWH: People living with HIV
