# Supplementary material for: Impact of the COVID-19 pandemic and the dynamic COVID-zero strategy on HIV incidence and mortality in China
Source: BMC Public Health. 2023 Feb 18;23:361. doi: 10.1186/s12889-023-15268-9 (PMC9938685; doi:10.1186/s12889-023-15268-9)
Supplement: Supplementary file 1 — Additional file 1: Supplementary Figure 1. Trends in the monthly incidence, mortality rates, and CFRs for HIV in China in 2015–2019, 2020, 2021, and 2022. Supplementary Figure 2. Scatter plot of the number of monthly reported HIV cases and the number of monthly reported COVID-19 cases in 2020–2022, China. [file 12889_2023_15268_MOESM1_ESM.docx]

**Supplementary Files**


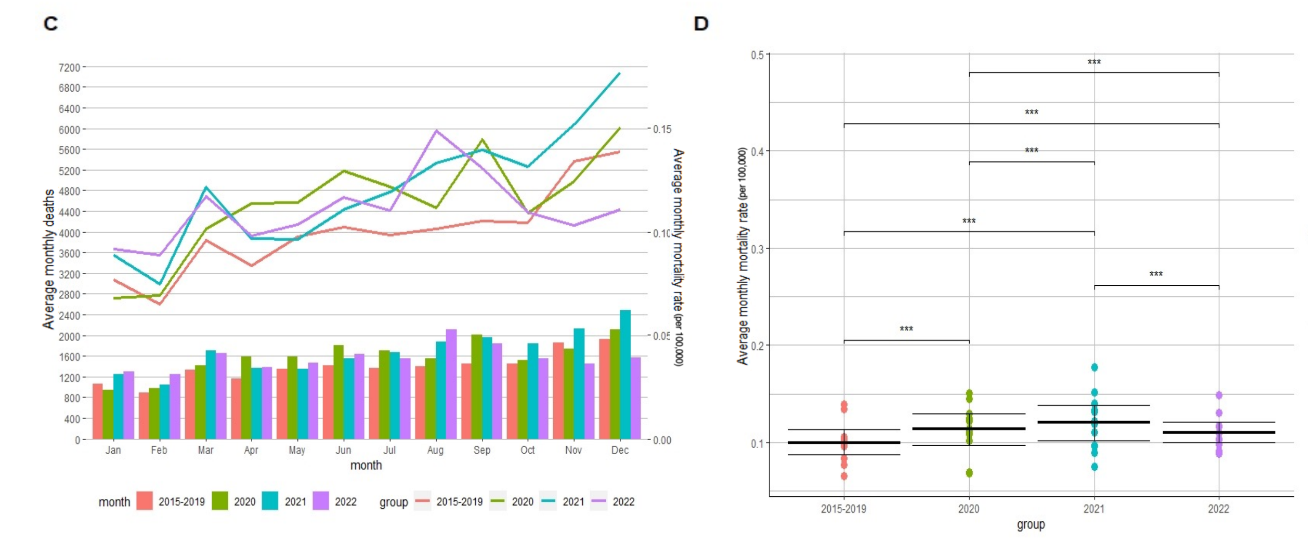

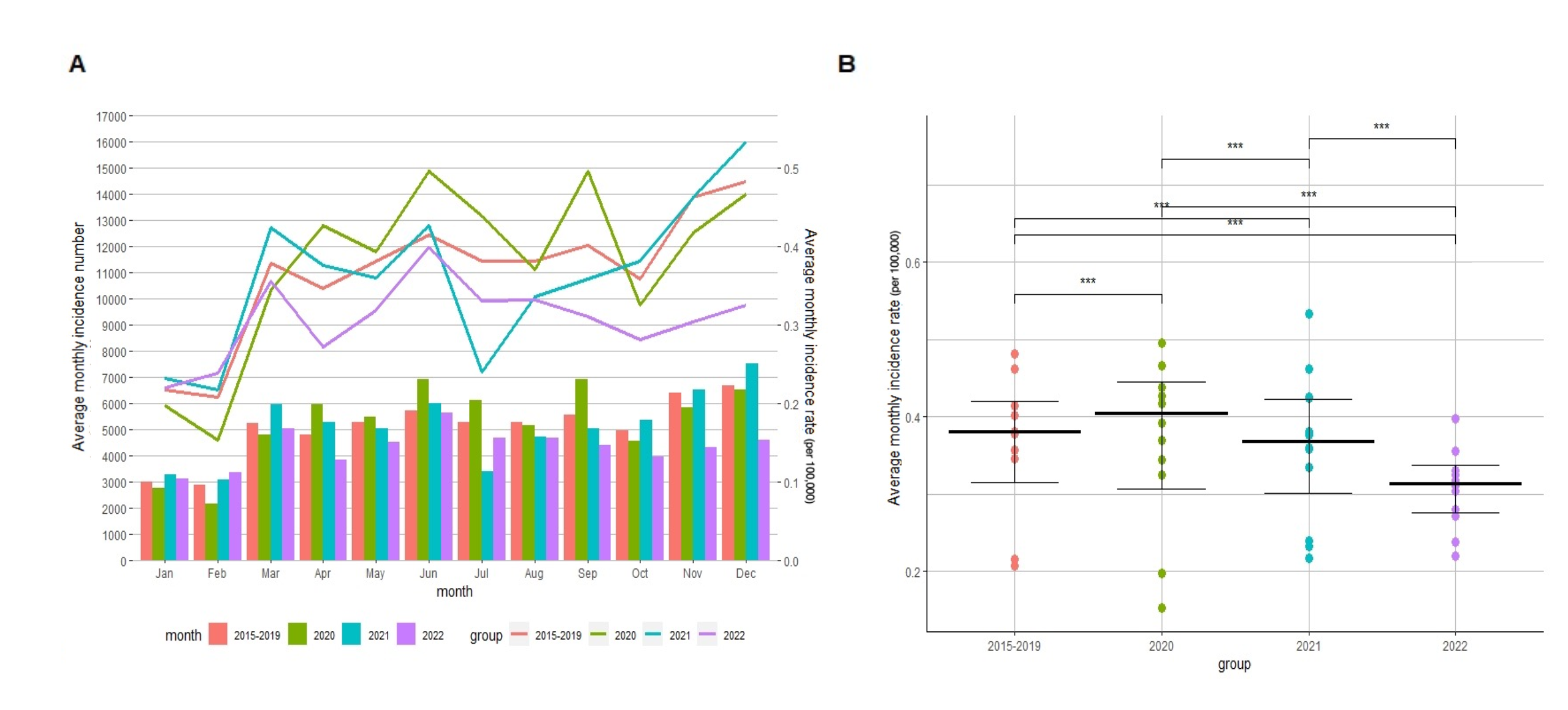


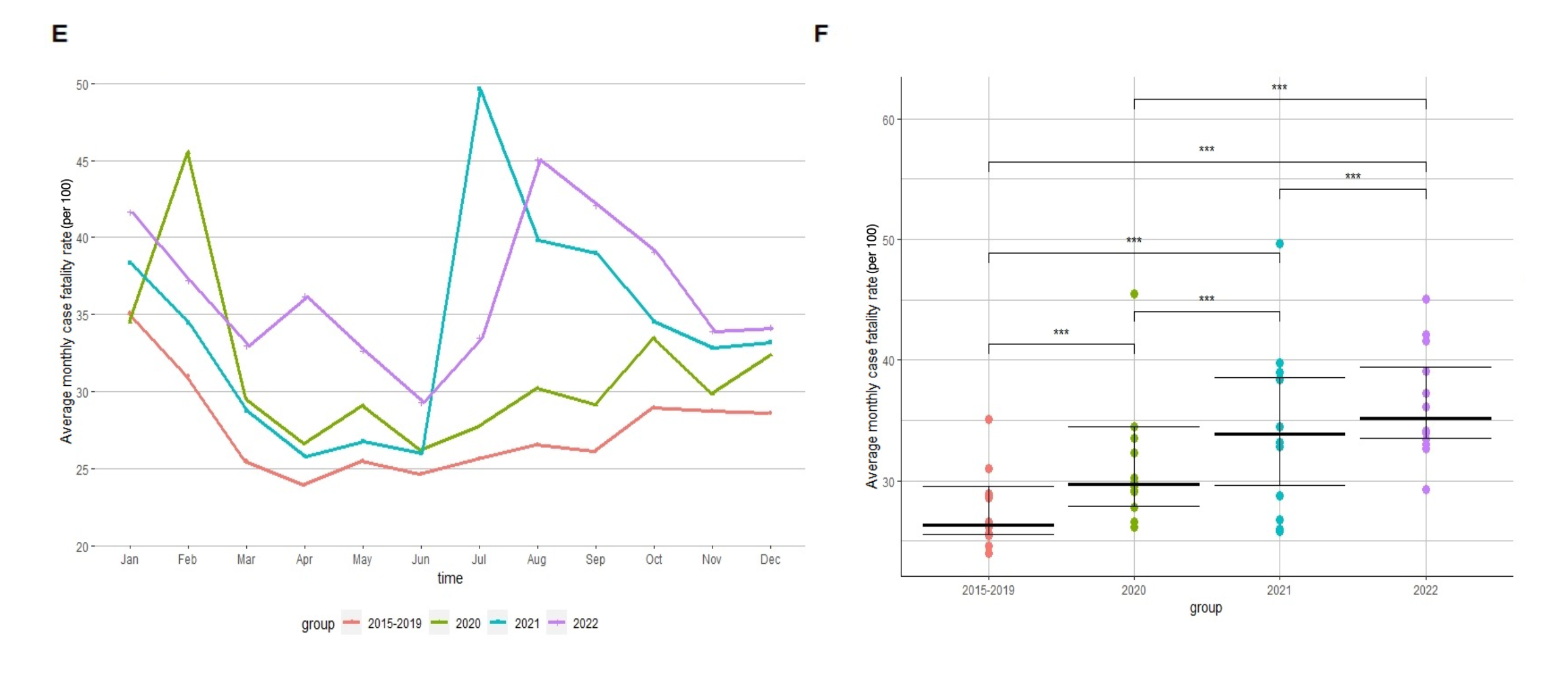
 **Supplementary Figure 1.** Trends in the monthly incidence, mortality rates, and CFRs for HIV in China in 2015–2019, 2020, 2021, and 2022.

Notes:

1. Figure 1A: Total number of reported HIV cases monthly and the monthly incidence of HIV in 2015–2019, 2020, 2021, and 2022;
2. Figure 1C: Total number of reported HIV deaths monthly and the monthly mortality rates of HIV in 2015–2019, 2020, 2021, and 2022;
3. Figure 1E: Monthly CFRs in 2015–2019, 2020, 2021, and 2022;
4. Figures 1B, 1D, and 1F: Comparisons of the yearly incidence, mortality rates, and CFRs for HIV in 2015–2019, 2020, 2021, and 2022 (***, *p* < 0.001; **, 0.001 < *p* < 0.01; *, 0.01 < *p* < 0.05). Black lines indicate P25, P50, and P75 for the upper and lower quartiles. Dots indicate the monthly incidence, mortality rates, and CFRs from January to December in different years.
5. Figures 1A,1B,1C, 1D, 1E, and 1F: 2015–2019 (red bar and red line), 2020 (green bar and green line),2021 (blue bar and blue line), and 2022 (purple bar and purple line).

**
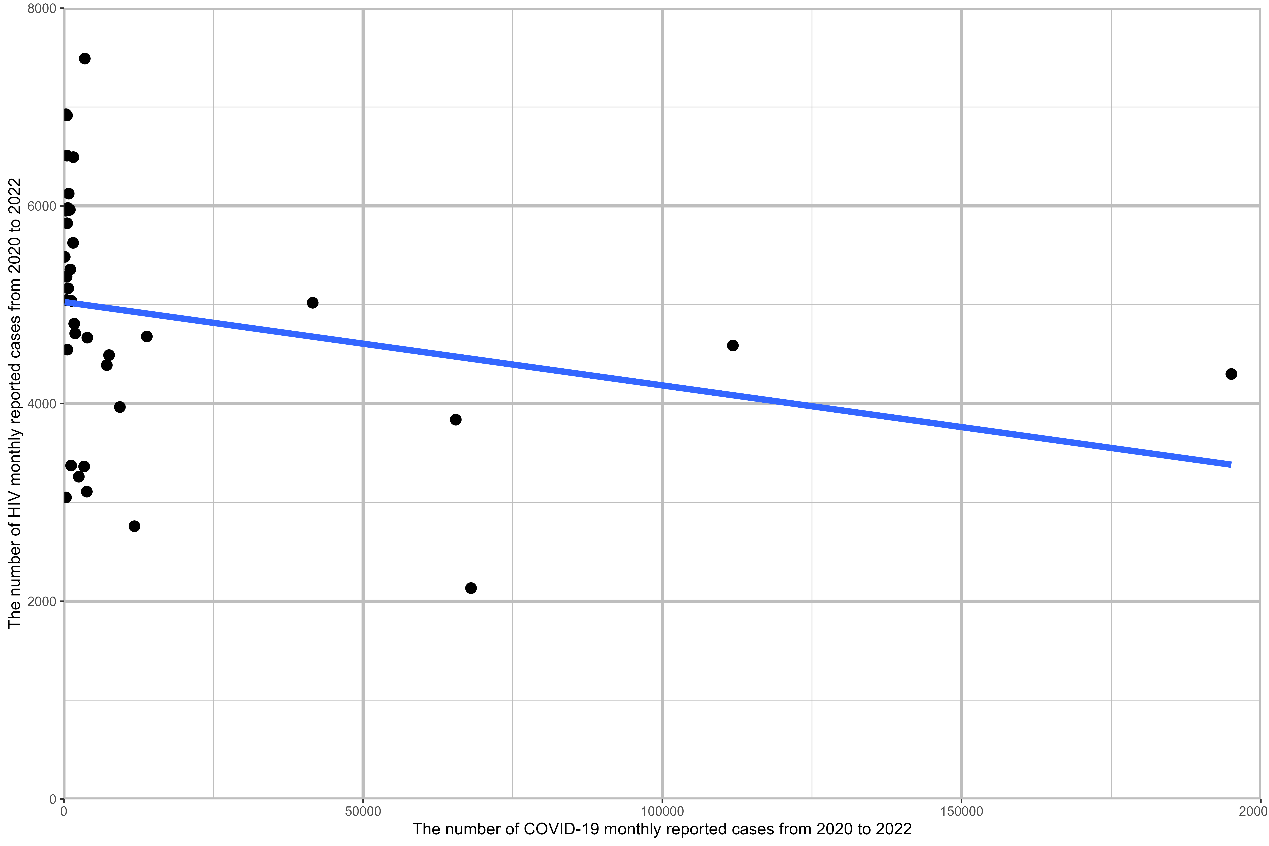
Supplementary Figure 2.** Scatter plot of the number of reported HIV cases monthly and the number of monthly reported COVID-19 cases in 2020–2022, China.

Notes: The line in Figure S2 is the regression line between the number of reported HIV cases monthly and the number of reported COVID-19 cases monthly in 2020–2022.The Wilcoxon rank test was applied to examine the correlation between the number of HIV cases monthly and the number of COVID-19 cases monthly.
